# Spontaneous onset of irregular collective oscillations in heterogeneous neural networks

**DOI:** 10.1186/1471-2202-12-S1-P206

**Published:** 2011-07-18

**Authors:** Stefano Luccioli

**Affiliations:** 1 CNR – Consiglio Nazionale delle Ricerche, Istituto dei Sistemi Complessi, I-50019, Italy; 2 INFN, Sez. Firenze, I-50019 Sesto Fiorentino, Italy

## 

More and more interest is devoted to identify the basic mechanisms underlying the emergence of nontrivial collective behaviors of large populations of neurons, one typical instance being the irregular background activity in the cerebral cortex [1]. Though accurate mathematical models have been developed for describing the microscopic activity of single neurons and of their synapsis [2], it is convenient to use relatively simple systems [3], under the assumption that they could be able to reproduce the relevant collective phenomena exhibited by realistic systems.

In this perspective, in Ref. [4] we numerically studied a network composed of *leaky integrate and fire* neurons [5], a class of simple single neuron models that exhibit a periodic spiking behavior in the absence of synaptic coupling. The coupling is the result of pulses that are sent across the system. The natural heterogeneity in the network is represented by the fact that different neurons have different spiking frequencies. Moreover we consider a delay between the pulse emission and its arrival.

The main result here discussed, and reported in Ref. [4], is that, upon increasing the coupling strength of the neurons, such a kind of network exhibits a transition to a *nontrivial collective dynamics*.

Relevant differences divide our findings from the transition observed in the Kuramoto model (KM) [6], a paradigmatic model for synchronization phenomena, describing the dynamics of an ensemble of coupled phase oscillators.

First, the model that we considered is not chaotic and it eventually converges to a periodic orbit. Moreover the transient time needed to approach some periodic orbit grows exponentially with the number of neurons. Thus implying that for large population of neurons the relevant dynamical regime is the “transient“, rather than the periodic orbit approached over astronomical time scales! This is an instance of “stable chaos” [7-9].

But the most striking difference with KM concerns the dynamical regime observed beyond the transition, in fact in this case the overall neural activity is not constant and not even simply periodically modulated, but exhibits irregular, seemingly chaotic, oscillations.

The mechanism here analyzed may contribute to the irregular background activity observed in the cerebral cortex.

## References

[B1] Destexhe A Rudolph M Paré D The high-conductance state of neocortical neurons in vivo Nature Neurosci 2003 4 739 751 10.1038/nrn1198 12951566

[B2] Gerstner W Kistler WM Spiking neuron models: Single neurons, populations, plasticity 2002 Cambridge University Press

[B3] Golomb G Hansel D Mato G Handbook of Biological Physics 2001 4 Amsterdam:Elsevier Science

[B4] Luccioli S Politi A Irregular Collective Behavior of Heterogeneous Neural Networks Phys Rev Lett 2010 105 1581041 1581044 10.1103/PhysRevLett.105.158104 21230943

[B5] Politi A Luccioli S Network Science: Complexity in Nature and Technology 2010 Heidelberg: Springer-Verlag

[B6] Kuramoto Y Chemical Oscillations, Waves, and Turbulence 1984 Berlin: Springer-Verlag

[B7] Politi A Torcini A Nonlinear dynamics and Chaos 2010 Heidelberg: Springer-Verlag

[B8] Jahnke S Memmesheimer RM Timme M Stable Irregular Dynamics in Complex Neural Networks Phys Rev Lett 2008 100 0481021 0481024 10.1103/PhysRevLett.100.048102 18352336

[B9] Zillmer R Brunel N Hansel D Very long transients, irregular firing, and chaotic dynamics in networks of randomly connected inhibitory integrate-and-fire neurons Phys Rev E 2009 79 0319091 03190913 10.1103/PhysRevE.79.031909 19391973

